# Comparative analysis of a 3D-printed videolaryngoscope versus the Macintosh model in adult airway management: a randomized clinical trial

**DOI:** 10.1016/j.bjane.2026.844757

**Published:** 2026-04-22

**Authors:** Florentino Fernandes Mendes, Liége Caroline Immich, Thiago Fagundes Gross, Luis Fernando Marcelino Braga, Andreia Gomes Aires, Elizandra Braganhol, Ramon Magalhães Mendonça Vilela, Francine Ullrich Carrazzoni dos Reis, Gustavo Zordan Piva, Victor Hugo Reinoso Gomez, Vinicius Hofstätter Rodrigues, Afonso Reguly, Leandro de Freitas Spinelli, Simone Schneider Amaral, Gisele Orlandi Introíni

**Affiliations:** a Universidade Federal de Ciências da Saúde de Porto Alegre (UFCSPA), Porto Alegre, RS, Brazil; b Programa de Pós-Graduação em Tecnologia da Informação e Gestão em Saúde, Universidade Federal de Ciências da Saúde de Porto Alegre (UFCSPA), Porto Alegre, RS, Brazil; c Santa Casa de Porto Alegre, Porto Alegre, RS, Brazil; d Laboratório de Inovação, Prototipagem, Educação Criativa e Inclusiva (LIPECIN), Universidade Federal de Ciências da Saúde de Porto Alegre (UFCSPA), Porto Alegre, RS, Brazil; e Programa de Pós-Graduação em Ciências da Reabilitação, Universidade Federal de Ciências da Saúde de Porto Alegre (UFCSPA), Porto Alegre, RS, Brazil; f Universidade Federal do Rio Grande do Sul (UFRGS), Porto Alegre, RS, Brazil

**Keywords:** 3D Printing, Additive Manufacturing, Airway Management, Intratracheal, Intubation

## Abstract

**Background and objectives:**

Video Laryngoscopy (VL) provides enhanced glottic visualization in airway management, but its high cost limits adoption. Three-Dimensional (3D) printing offers a cost-effective alternative for producing VL devices. This randomized trial compared a 3D-printed Videolaryngoscope (VL3D) with the standard Macintosh Laryngoscope (MAC) during tracheal intubation in adults.

**Methods:**

This was a randomized, controlled, and assessor-blinded clinical trial. The primary outcome was intubation difficulty, assessed using the Intubation Difficulty Scale (IDS). Secondary outcomes included glottic visualization (Cormack-Lehane grade) and Intubation Time (IT). The final analysis followed a modified intention-to-treat principle.

**Results:**

This study included 52 patients scheduled to undergo general anesthesia with tracheal intubation. Baseline characteristics were comparable between groups. The analysis included 49 patients (VL3D, n = 26; MAC, n = 23). Categorical analysis of the primary outcome showed a higher incidence of moderate-to-severe intubation difficulty in the VL3D group (23.1% vs. 0%, p = 0.032), although median IDS scores were statistically similar. In relation to secondary outcomes, the VL3D provided significantly superior glottic visualization (Cormack-Lehane grade I: 72.0% in the VL3D group vs. 33.3% in the MAC group, p = 0.002). Conversely, the VL3D was associated with a significantly longer intubation time (median 70 seconds vs. 40 seconds, p = 0.005).

**Conclusions:**

The VL3D demonstrated superior glottic visualization but correlated with significantly longer intubation duration and greater procedural difficulty. These findings suggest that additional specialized training or design optimization is necessary before widespread clinical implementation.

## Introduction

Evidence from randomized trials and systematic reviews indicates that video-assisted techniques yield better glottic exposure and improved first-pass success rates than direct laryngoscopy.^1^, ^2^, ^3^ However, despite these visual benefits, no statistically significant differences have been consistently observed in terms of intubation duration or the incidence of airway-related injuries, including trauma to the lips, gums, teeth, or pharyngeal structures. While videolaryngoscopes optimize management in anatomically complex cases, their implementation is often hindered by high procurement costs and restricted availability in low-resource environments.

A promising alternative to conventional industrial manufacturing is the use of Industry 4.0 technologies, particularly Computer Numerical Control (CNC) systems and additive manufacturing (3D printing). These tools allow for the low-cost, small-scale production of customized medical devices. One study employed 3D printing to fabricate a laryngoscope blade compatible with a borescope, which served as both the light source and video feed, highlighting the potential for low-cost, anatomically tailored blade designs.^4^ Another innovation combined a Universal Serial Bus (USB) endoscopic camera with a laryngoscope, utilizing a standard personal computer for real-time image display.^5^

Consistent with this approach, our research group developed a customized, 3D-printed Videolaryngoscope (VL3D) for adult and pediatric use. The device's functional reliability and usability were previously validated in preclinical settings. Mechanical testing demonstrated adequate structural integrity, while simulation-based evaluations using airway mannequins confirmed ergonomic handling and optimal glottic visualization. Furthermore, the video output was consistently rated as high-quality by independent evaluators.^6^

Building upon these promising preclinical and simulation-based findings, this randomized clinical trial was designed to assess the performance of the VL3D device in comparison with the conventional Macintosh laryngoscope. The primary outcome was the level of difficulty encountered during intubation (measured by the Intubation Difficulty Scale). Secondary outcomes included glottic visualization (Cormack-Lehane grade), intubation time, procedural efficiency, and the incidence of adverse events, including bleeding, dental trauma, mucosal injury, and lip damage.

## Material and methods

### *Study location*

This research was conducted at the Surgical Center of the Santa Casa de Porto Alegre (SCPA), in collaboration with the Outpatient Preoperative Evaluation Clinic (OPEC) and the Santa Casa Anesthesia Service (SCAS).

### *Ethical considerations*

This study included 52 patients scheduled to undergo general anesthesia with tracheal intubation. All procedures were conducted in accordance with Resolution n°466/2012 of the Brazilian National Health Council and the ethical principles of the World Medical Association’s Code of Ethics. The study protocol was approved by the Institutional Research Ethics Committee (CAAE: 58735522.8.0000.5335), and the trial was registered at the Brazilian Clinical Trials Registry (ReBEC) under number RBR-9t36r35 before the first patient enrollment. The manuscript adheres to the Consolidated Standards of Reporting Trials (CONSORT) guidelines, and written informed consent was obtained from all participants.

### *Trial design*

This study was a randomized, controlled, and assessor-blinded clinical trial designed to compare the VL3D device with the Macintosh laryngoscope, which is widely regarded as the gold standard in airway management.

The randomization sequence was generated using the randomizeR package in R software by an independent investigator not involved in recruitment or data collection. To ensure allocation concealment, participants were assigned to study groups using sequentially numbered, opaque, sealed envelopes. These envelopes were opened by the attending anesthesiologist only after the participant had been formally enrolled and baseline data were obtained, thereby minimizing selection bias.

### *Inclusion criteria*

Eligible participants were adult patients (≥ 18 years), scheduled for surgical procedures under general anesthesia requiring tracheal intubation. All were classified as ASA physical status I to IV and provided written informed consent.

### *Exclusion criteria*

Patients were excluded from the study if they were scheduled for emergency surgical procedures; undergoing upper airway, head and neck, or orthognathic surgery; or required planned nasotracheal intubation. Additional exclusion criteria included a history of cervical spine surgery, the presence of cervical disc injuries or fractures, or the use of a cervical collar. Furthermore, individuals with a Body Mass Index (BMI) ≥ 35 kg.m^−2^, those diagnosed with Type 1 Diabetes Mellitus, or patients with syndromic conditions or head and neck trauma that could interfere with standard intubation techniques were also ineligible.

### *Primary and secondary outcome measures*

#### Primary outcome

The primary outcome of this study was the intubation difficulty, quantified by the Intubation Difficulty Scale (IDS).

#### Secondary outcomes

Secondary outcomes included glottic visualization, as assessed by the Cormack-Lehane (C-L) grade, Intubation Time (IT), procedural efficiency, and the incidence of adverse events: bleeding, dental trauma, mucosal injury, and lip damage.

### *Anesthetic management*

Intraoperative monitoring included electrocardiography with leads DII and V5, pulse oximetry, capnography, Noninvasive Blood Pressure (NIBP), and neuromuscular blockade assessment using Train-of-Four (TOF) monitoring. Anesthetic management was standardized across participants. Fentanyl was administered at an initial bolus dose of 5 µg.kg^−1^, with supplemental doses provided as needed and recorded at the end of the procedure. Propofol was used for induction at an initial dose of 2 mg.kg^−1^, titrated according to clinical requirements. The administration of inhalational anesthetic (sevoflurane) was documented as volume percent, while oxygen consumption was measured in liters per minute over the total period of use. Rocuronium was employed as the neuromuscular blocking agent at a dose of 0.6 mg.kg^−1^ (equivalent to 2× ED_95_), with additional boluses of one-fifth the induction dose administered at the anesthesiologist’s discretion. Other adjuvant drugs used during induction and maintenance of anesthesia were also administered based on clinical judgment.

### *Airway management: Standardization of intubation technique*

All patients were positioned on a 10 cm rigid cushion to optimally align the laryngeal and tracheal axes. Preoxygenation with FiO_2_ > 80% for ≥ 3 minutes preceded anesthetic induction. Intubation began once TOF = 0 (adductor pollicis), with a 15-second pause between stimuli.

Intubation time was recorded from TOF zero to tracheal sealing by a cuff inflated. Laryngoscopy was performed with a VL3D (model LIPECIN)^6^ or Macintosh laryngoscope (blade sizes 4‒5), according to the randomized allocation. In the event of intubation difficulty, the attending anesthesiologist was permitted to switch to the alternative device, guided by the Intubation Difficulty Scale (IDS). The internal diameter of the endotracheal tube was chosen at the discretion of the anesthesiologist, and intubation was performed using a standardized, single-use tube introducer.

### *Intubator training with the VL3D*

Prior to patient enrollment, all anesthesiologists responsible for airway management ‒ each with 3 to 6 years of specialty experience ‒ underwent structured training with the VL3D at the Institutional Simulation Center (UFCSPA). To ensure proficiency, each participant completed a minimum of 20 supervised intubations on simulation mannequins featuring varying airway difficulty levels.

### *Airway assessment and variable definitions*

Airway assessment followed standardized protocols based on validated predictors of difficult intubation. The Modified Mallampati Test (MMT) was conducted with the patient seated, head in a neutral position, and mouth opened to its maximum. Scores ranged from Class I to IV, with Classes III and IV considered predictive of difficult intubation. Thyromental Distance (TMD) was measured from the mentum to the thyroid cartilage with the head fully extended; distances ≤ 6.0 cm were classified as high risk. Head and neck mobility was evaluated by measuring the angle between the mandible and cervical spine during maximal extension. Dental protrusion was classified visually as absent, moderate (0–0.5 cm), or severe (> 0.5 cm). Interincisor distance was measured with the mouth maximally opened and recorded in centimeters.

Mandibular mobility and dental relationship were further assessed using the Upper Lip Bite Test (ULBT), in which the lower incisors' ability to contact the upper lip vermilion line was categorized as Class I (complete cover), Class II (partial cover), or Class III (unable to reach). To ensure consistency in measurement, all evaluators underwent specific training, and measuring instruments were calibrated. The Airway Assessment Score (AAS) was calculated by assigning numerical values to the results of the above tests, according to predefined criteria. A total score above 6 was considered predictive of a difficult airway (Table 1).^7^

**Table 1:** Airway Assessment Score (AAS).

Variables	Points		
	0	1	2
MMP	Class I	Class II	Class III – IV
TMD (cm)	> 6.5	6 – 6.5	< 6.0
Head / Neck mobility (°)	> 90	90	< 90
BMI (kg.m^−2^)	< 25	≥ 25	–
Protruded teeth	No	Moderate	Severe
Interincisor distance	> 5	4 – 5	< 4
ULBT	Class I	Class II	Class III

MMP, Modified Mallampati classification; TMD, Thyromental Distance; BMI, Body Mass Index; ULBT, Upper Lip Bite Test. Note: Scores above 6 points are considered predictive of difficult intubation.

### *Intubation difficulty assessment*

The Intubation Difficulty Scale (IDS) was employed during the procedure to objectively quantify intubation difficulty. This assessment was based on seven parameters: the number of intubation attempts, the amount of operators involved, the use of alternative techniques, the Cormack-Lehane (C-L) grade, the application of increased lifting force, the use of external laryngeal pressure, and the position of the vocal cords. Scores were recorded immediately after intubation by the performing anesthesiologist. Total IDS scores were categorized as follows: 0 = Easy, 1–5 = Mild difficulty, and >5 = Moderate to severe difficulty.

The Cormack-Lehane grade was documented during initial laryngoscopy without external laryngeal pressure. For analytical purposes, grades I and II were considered easy, and grades III and IV were classified as difficult. The study analyzed correlations between C-L grade, AAS, and IDS, with continuous and categorical analyses of IDS scores (Table 2).^8^

**Table 2:** Intubation Difficulty Scale (IDS).

Variables	Points
N1 – Each intubation attempt adds	1
N2 – Each additional operator adds	1
N3 – Each alternative technique adds	1
N4 – Apply Cormack-Lehane (C-L) grade for first attempt	0 – 1
N5 – Increased lifting force required for intubation add 1 point	0 – 1
N6 – External laryngeal pressure to improve C-L?	1
N7 – Position of the vocal cords during intubation	0 – 1

Notes: N3 – One point is added if any alternative technique is required, including patient repositioning, modification of equipment (e.g., blade, endotracheal tube, or the addition of a stylet), alteration of the intubation route (nasotracheal or orotracheal), or the use of devices such as fiberoptic scopes or laryngeal mask airways; N4 – A Grade I view on the first attempt corresponds to 0, while any deviation from this grade adds 1-point; N5 – Normal lifting force corresponds to 0. Increased force during laryngoscopy adds 1-point; N6 – Application of external laryngeal pressure to improve glottic visualization adds 1-point; N7 – Vocal cord position is scored as 0 for abduction and 1 for adduction. An IDS score of 0 indicates easy intubation; IDS 1–5 indicates mild difficulty; and IDS >5 indicates moderate-to-severe difficulty.

### *Biological safety and processing of devices*

The VL3D devices underwent a standardized sterilization protocol. This process included immediate pre-cleaning, manual washing with a mild detergent and non-abrasive materials, drying using disposable cloths and compressed air, followed by inspection and packaging. Packaging utilized a cellulose-free barrier suitable for hydrogen peroxide processing. Sterilization was performed using hydrogen peroxide. Post-sterilization, the devices were stored in a clean, dry environment, shielded from light exposure and excessive handling. The validated shelf life of the sterilization process was one year. Furthermore, all units were subjected to microbiological testing conducted by the institutional laboratory.

### *Borescope cameras*

Borescope cameras were subjected to a separate process: they were manually washed, disinfected with 70% alcohol, dried, and stored in sealed, labeled plastic bags under the same handling conditions as the VL3D units. The disinfection efficacy was considered valid for 30 days. In contrast, conventional Macintosh laryngoscopes were processed and sterilized in accordance with the established institutional protocol.

### *VL3D production and traceability*

The VL3D device is a fully reproducible, low-cost solution (approx. $12 USD), developed based on a well-established blade geometry, such as the C-MAC configuration. Its construction utilized conventional Fused Deposition Modeling (FDM) technology and incorporated a standard camera module, an LED light source, and a dedicated display unit.

The design initiative is licensed under the Creative Commons Attribution-Noncommercial-No Derivatives (CC BY-NC-ND) license, permitting non-commercial redistribution under the condition that the device is not altered or used for commercial gain. The complete design was previously published and validated by our research group.^6^ All requisite STL files, comprehensive assembly instructions, and the full Bill of Materials (BOM) are publicly accessible in the supplementary materials of the original publication, thereby ensuring high reproducibility across intrahospital makerspaces.

A total of 16 VL3D units were produced in the institutional prototyping laboratory. To ensure traceability and aseptic handling, each device was labeled with a unique identifier, stored in sealed packaging, and handled aseptically. A designated researcher, independent of the clinical phases of the study, was responsible for sterilization, biological safety monitoring, and reuse tracking.

### *Adverse event monitoring*

The following adverse events were monitored and recorded as binary outcomes: bleeding, dental trauma, mucosal injury, and lip damage.

### *Statistical analysis*

Data were entered into Microsoft Excel and analyzed using SPSS version 20.0. The final analysis followed a Modified Intention-To-Treat (mITT) principle. Although 52 patients were initially randomized, three patients in the MAC group were excluded from the final analysis due to post-randomization protocol deviations (failure to meet inclusion criteria discovered after allocation), resulting in a final sample of 49 patients (VL3D, n = 26; MAC, n = 23), as detailed in the CONSORT flow diagram. Categorical variables were summarized as frequencies and percentages and compared using the Chi-square test with Yates' correction or Fisher's exact test, as appropriate. Categorical analysis of the primary outcome (IDS) was also performed. Pairwise comparisons of proportions were conducted with the z-test when applicable.

Normality of quantitative data was assessed with the Shapiro-Wilk test. Variables that were normally distributed were expressed as mean ± standard deviation and compared using the Student's *t*-test for independent samples. Non-normally distributed variables were presented as median and interquartile range (25^th^–75^th^ percentiles) and analyzed with the Mann-Whitney *U*-test.

The Intubation Time (IT) was additionally analyzed using the Kaplan-Meier method to estimate the probability of successful intubation over time, with groups compared via the log-rank test. To supplement this, a Cox proportional hazards regression model was applied to calculate the Hazard Ratio (HR) and its 95% Confidence Interval, evaluating the relative rate of intubation success between the VL3D and Macintosh groups.

### *Sample size and power calculation*

A minimum sample size of 46 patients (23 per group) was initially estimated based on a conservative a priori calculation for the primary outcome (IDS). This was calculated to detect an expected two-point difference in Intubation Difficulty Scale (IDS) scores (an effect considered clinically relevant). The calculation assumed a standard deviation of approximately 2 points, a significance level (α) of 0.05, and 80% power. To compensate for potential losses due to follow-up or protocol deviations, an additional 10% was added, resulting in a total sample of 52 patients to be enrolled. This calculation was performed using the PSS Health online tool.^9^

## Results

**Figure 1:**
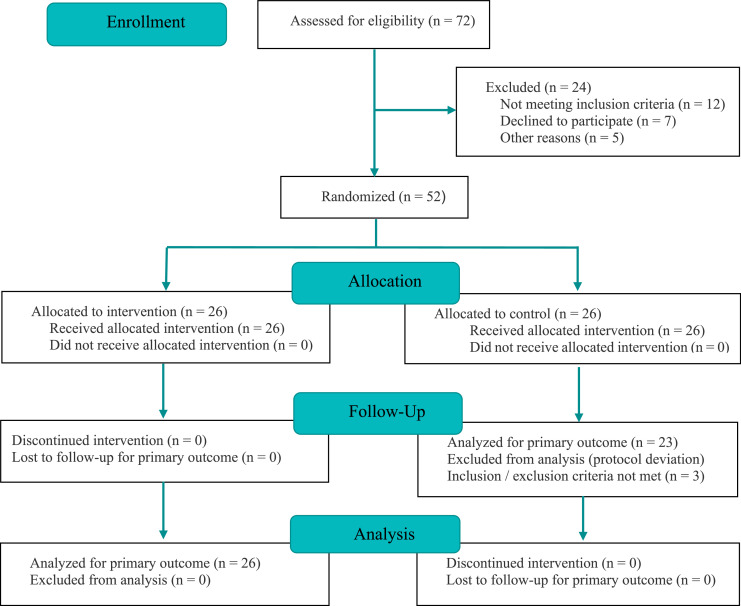
CONSORT flow diagram illustrating the enrollment allocation, follow-up, and analysis of patients randomized to receive intubation with VL3D versus the MAC.

A total of 52 adult patients undergoing general anesthesia were initially enrolled in the study. The final analysis followed a modified intention-to-treat principle, including 49 participants: 26 in the VL3D group and 23 in the MAC group. Following randomization, three patients from the MAC group were excluded due to protocol deviations (failure to meet inclusion criteria discovered after allocation). These findings are detailed in the CONSORT flow diagram (Fig. 1). No statistically significant differences were observed between the groups in terms of sociodemographic or clinical characteristics (Table 3).

**Table 3:** Comparison between different sociodemographic and clinical groups.

Characteristics	VL3D Group		MAC Group		p
	n	Descriptive measures	n	Descriptive measures	
Gender, n (%)	26		23		0.405^a^
Female			14 (53.8)		16 (69.6)
Male			12 (46.2)		7 (30.4)
Age, mean ± SD	26	58.2 ± 13.9	23	61.9 ± 16.2	0.414^b^
Surgical complexity n (%)	26		22		0.100^a^
Low			4 (15.4)		2 (9.1)
Medium			11 (42.3)		16 (72.7)
High			11 (42.3)		4 (18.2)
Weight, mean ± SD	26	73.2 ± 15.3	23	75.3 ± 15.3	0.641^b^
Height, mean ± SD	26	1.65 ± 0.09	23	1.66 ± 0.10	0.689^b^
BMI, mean ± SD	26	26.8 ± 4.3	23	27.4 ± 5.2	0.662^b^
ASA – PS, n (%)	25		22		0.999^a^
I			2 (8.0)		2 (9.1)
II			17 (68.0)		14 (63.6)
III			6 (24.0)		6 (27.3)

BMI, Body Mass Index; ASA-PS, American Society of Anesthesiologist Physical Status Classification System; SD, Standard Deviation.

a Chi-Square test with Yates correction or Fisher's exact test.

b Student's *t*-test for independent samples.

### *Primary outcome*

The primary outcome, assessed by the Intubation Difficulty Scale (IDS), showed that median scores were statistically similar between groups. However, categorical analysis revealed a significantly higher incidence of moderate-to-severe intubation difficulty (IDS > 5) in the VL3D group (23.1%) compared to the MAC group (0%) (p = 0.032). These results are detailed in Table 4.

**Table 4:** Comparison of outcomes between groups.

	VL3D Group		MAC Group		p
	n	Descriptive measure	n	Descriptive measure	
**AAS, mean ± SD**	26	5.04 ± 2.74	23	4.48 ± 2.7	0.479*
**Categories AAS, n (%)**	26		23		
≤ 6 points		19 (73.1)		17 (73.9)	0.999⁎⁎
> 6 points		7 (26.9)		6 (26.1)	
**Cormack-Lehane grade, n (%)**	25		21		0.002⁎⁎
1		18 (72.0)^b^		7 (33.3)^a^	
2		7 (28.0)		6 (28.6)	
3		0 (0)^b^		6 (28.6)^a^	
4		0 (0)		2 (9.5)	
**IT (sec), median (IQR)**	26	70 (40–105)	23	40 (30–43)	0.005⁎⁎⁎
**IDS, median (IQR)**	26	2 (0–5)	23	1 (0–3)	0.240⁎⁎⁎
**Categories IDS, n (%)**	26		22		0.032⁎⁎
0 – Easy		10 (38.5)		8 (36.4)	
0–5 ‒ Mild difficulty		10 (38.5)		14 (63.6)	
> 5 – Moderate to severe difficulty		6 (23.1)^b^		0 (0%)^a^	

AAS, Airway Assessment Score; SD, Standard Deviation; IT, Intubation Time (sec); IQR, Interquartile Range (percentiles 25–75); IDS, Intubation Difficulty Scale.

⁎ Student's *t*-test for independent samples.

⁎⁎ Chi-Square test with Yates correction or Fisher's exact test.

⁎⁎⁎ Mann-Whitney test.

a,b Different superscript letters indicate significant.

### *Secondary outcomes*

No statistically significant differences were observed between the groups in relation to the Airway Assessment Score (AAS), whether analyzed as a continuous variable (p = 0.479) or dichotomized into scores below and above 6 points (p = 0.999). Regarding glottic visualization, the Cormack-Lehane classification revealed a statistically significant difference between groups (p = 0.002). A significantly higher proportion of VL3D patients were classified as Cormack-Lehane grade I (72.0%) compared to MAC patients (33.3%) (p = 0.002). Conversely, grade III classifications were more frequent in the MAC group (28.6%) and entirely absent in the VL3D group (0%) (Table 4).

### *Intubation time and adverse events*

**Figure 2:**
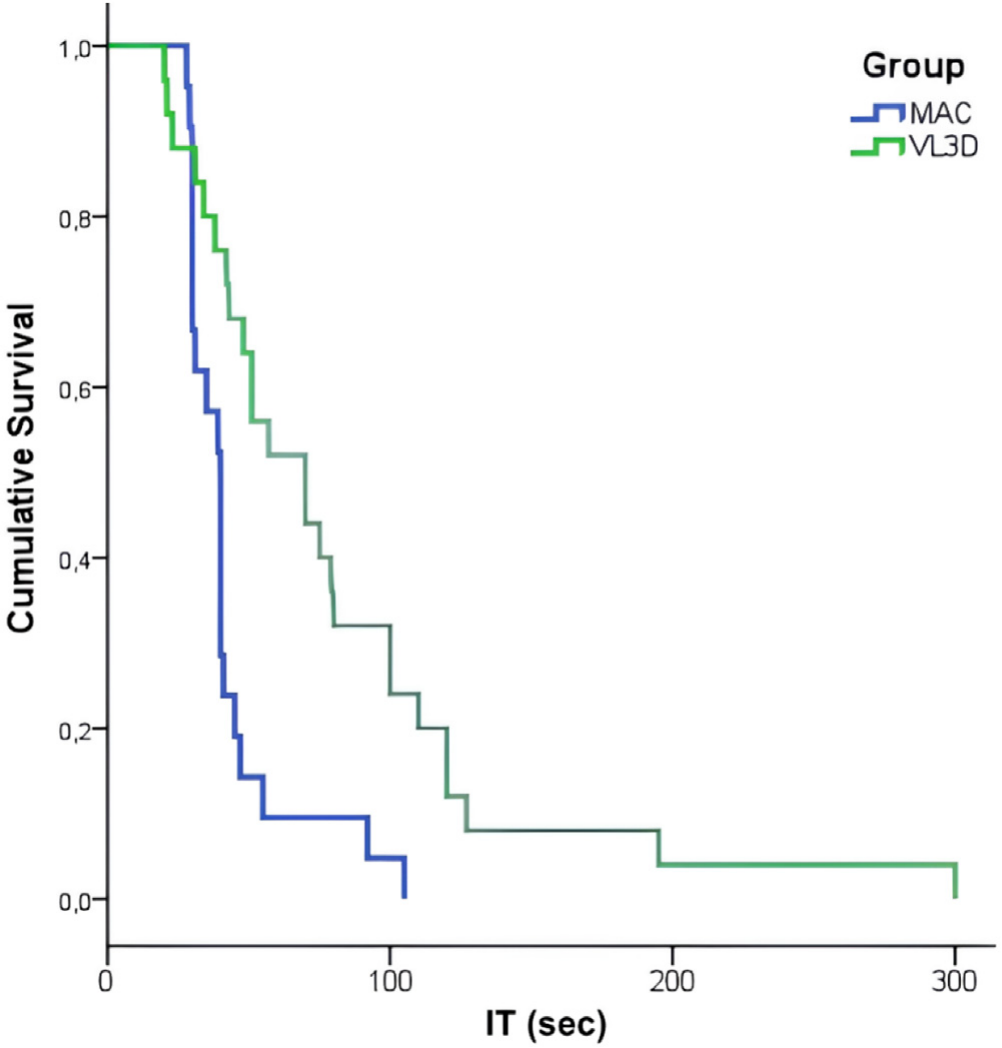
Kaplan-Meier curves for the probability of successful intubation over time. The Macintosh (MAC) group (blue line) demonstrated a significantly higher probability of rapid intubation success compared to the VL3D group (green line) (log-rank test, p = 0.001). IT, Intubation Time (sec).

The Kaplan-Meier analysis demonstrated a significantly lower probability of rapid successful intubation over time for the VL3D group compared to the MAC group (log-rank test, p = 0.001; Fig. 2). The estimated median time for successful intubation was 70 seconds (95% CI 39.1‒100.8) for the VL3D group and 40 seconds (95% CI 36.6–43.3) for the MAC group. Cox proportional hazards regression confirmed these findings, indicating that the MAC group achieved successful intubation at a significantly higher rate per unit of time than the VL3D group. No significant differences were found in the incidence of adverse events (bleeding, dental trauma, or mucosal injury) between the VL3D and MAC groups.

## Discussion

In this randomized controlled trial, we investigated the performance of the VL3D in comparison with the traditional Macintosh laryngoscope for tracheal intubation in patients undergoing general anesthesia. Our findings demonstrate that the VL3D was associated with higher procedural difficulty and longer intubation times, despite providing improved glottic visualization. The primary outcome (IDS) revealed a higher frequency of moderate-to-severe intubation difficulty in the VL3D group compared to the MAC group in categorical analysis.

Videolaryngoscopes (VLs) offer several advantages over Direct Laryngoscopes (DLs), including enhanced glottic visualization, improved coordination between operator and assistant, a shorter learning curve, ease of use for novice practitioners, a wider field of view, reduced intubation failure rates, and minimal need for head or neck manipulation.^10^ These benefits have contributed to the growing adoption of VLs not only in cases of anticipated difficult airway management but also as replacements for DLs in routine clinical practice.

Commercially available VLs vary considerably in blade design. Some models retain the general shape of the Macintosh blade, while others feature more pronounced angulation.

The VL3D used in this study featured a curved blade that consistently provided an adequate view of the glottis. Regarding our secondary outcomes, the VL3D consistently provided an adequate view of the glottis. Notably, all patients in the VL3D group were classified as Cormack-Lehane Grade I or II, whereas the MAC group included patients with Grades III and IV classifications. This distinction is clinically relevant, as poor glottic visualization (Grades III or IV) is associated with a twofold increase in airway-related complications, especially in non-operating room environments.^11^

It is important to recognize that patient safety during airway management is multifactorial. Although VLs represent a significant advancement in airway visualization, their contribution to safety is maximized only when integrated into comprehensive, well-structured airway management protocols.^12^

Although the VL3D provided enhanced glottic visualization, it is crucial to note that this did not translate into an easier intubation process. The implementation of Kaplan-Meier curves and Cox regression analysis provided a robust reinforcement of our findings regarding the intubation time. These methods highlighted a lower probability of rapid intubation success with the VL3D compared to the Macintosh laryngoscope. This suggests that while the VL3D model offers superior visualization, it may currently present additional difficulties in clinical performance, particularly in emergency situations or difficult airway management where time is a critical factor. These results reinforce that the VL3D model requires further design optimization and more extensive clinical testing in real-life scenarios before it can be recommended for time-sensitive airway interventions. This outcome may be partially attributed to disparities in operator experience between the two devices. The anesthesiologists participating in the study had between 3 to 6 years of experience/training in the specialty and had greater familiarity with the Macintosh laryngoscope, which likely contributed to the increased challenges observed with the VL3D. This discrepancy in prior training represents a significant methodological limitation of the study.

It is also plausible that the VL3D prototype used in this study may delay intubation in straightforward cases, although performance may vary across different VL models. Blade design may confer specific advantages depending on clinical context, such as intubation in obese patients, anticipated difficult airways, emergency settings, or when performed by less experienced clinicians.^2^

### *Study limitations and applicability*

It is worth noting that randomization produced well-balanced groups in terms of demographic and clinical characteristics. Furthermore, no statistically significant differences were found in the Airway Assessment Score (AAS), whether analyzed as a continuous or categorical variable, suggesting a comparable baseline risk of difficult airway between groups. These findings strengthen the internal validity of our results and support the interpretation that increased intubation difficulty in the VL3D group was not due to intrinsic patient-related airway complexity. A significant limitation of this trial is the exclusion of patients with predictors of difficult airway, such as those with a Body Mass Index (BMI) greater than 35 kg.m^−2^. Given that videolaryngoscopes are particularly recommended for difficult airways, it is highly plausible that, in this specific subgroup of patients (e.g., morbidly obese), the VL3D would demonstrate superior performance compared to the conventional Macintosh blade. Therefore, the findings regarding procedural time and difficulty are primarily applicable to patients with non-difficult airways.

Additional limitations include the lack of blinding for the intubating clinicians, who were aware of group assignments. Although blinding was maintained throughout other phases of the study, the inability to blind the clinician responsible for the intubation may have introduced bias.

## Conclusion

In conclusion, the VL3D videolaryngoscope demonstrated superior glottic visualization but was associated with increased intubation difficulty and longer procedural times compared to the Macintosh laryngoscope. These results underscore the importance of structured training and suggest potential for further optimization of the device before clinical implementation. Additional studies are being conducted by our research group to optimize the design and validate the long-term clinical feasibility of 3D-printed laryngoscopes.

## Conflicts of interest

The authors declare no conflicts of interest.

## References

[1] Lewis S.R., Butler A.R., Parker J., Cook T.M., Smith AF. (2022). Videolaryngoscopy versus direct laryngoscopy for adult patients requiring tracheal intubation. Cochrane Database Syst Rev.

[2] Hansel J., Rogers A.M., Lewis S.R., Cook T.M., Smith AF. (2022). Videolaryngoscopy versus direct laryngoscopy for adults undergoing tracheal intubation: A Cochrane systematic review and meta-analysis update. Br J Anaesth.

[3] Prekker M.E., Driver B.E., Trent S.A., Resnick-Aultet D. (2023). DEVICE Investigators and the Pragmatic Critical Care Research Group. Video versus direct laryngoscopy for tracheal intubation of critically Ill adults. N Engl J Med.

[4] Fonternel T., van Rooyen H., Joubert G., Turton E. (2023). Evaluating the usability of a 3D-printed video laryngoscope for tracheal intubation of a manikin. Med Devices.

[5] Karippacheril J.G., Cong ML. (2016). Videolaryngoscopy using an Android smartphone: A direct digital technique. Indian J Anaesth.

[6] Mendes F.F., Spinelli L.F., Dutra P.A.S., de Braga E.S. (2023). Three-dimensional printed laryngoscopes as allies against COVID-19. 3D Print Addit Manuf.

[7] Seo S.H., Lee J.G., Yu S.B., Kim D.S. (2012). Predictors of difficult intubation defined by the Intubation Difficulty Scale (IDS): predictive value of 7 airway assessment factors. Korean J Anesthesiol.

[8] Adnet F., Borron S.W., Racine S.X., Clemessy J.L. (1997). The Intubation Difficulty Scale (IDS): proposal and evaluation of a new score characterizing the complexity of endotracheal intubation. Anesthesiology.

[9] Borges R.B., Mancuso A.C.B., Camey S., Leotti V.D. (2021). Power and Sample Size for Health Researchers: uma ferramenta para cálculo de tamanho amostral e poder do teste voltado a pesquisadores da área da saúde. Clin Biomed Res.

[10] Sakles J.C., Rodgers R., Keim SM. (2008). Optical and video laryngoscopes for emergency airway management. Intern Emerg Med.

[11] Martin L.D., Mhyre J.M., Shanks A.M., Tremper K.K. (2011). Kheterpal S. 3,423 emergency tracheal intubations at a university hospital: airway outcomes and complications. Anesthesiology.

[12] Paolini J.B., Donati F., Drolet P. (2013). Review article: video-laryngoscopy: another tool for difficult intubation or a new paradigm in airway management?. Can J Anaesth.

